# Functional Compartmentalization of the Contribution of Hippocampal Subfields to Context-Dependent Extinction Learning

**DOI:** 10.3389/fnbeh.2019.00256

**Published:** 2019-11-14

**Authors:** Marta Méndez-Couz, Jana M. Becker, Denise Manahan-Vaughan

**Affiliations:** Department of Neurophysiology, Medical Faculty, Ruhr University Bochum, Bochum, Germany

**Keywords:** appetitive learning, extinction learning, hippocampus, immediate early gene, spatial memory

## Abstract

During extinction learning (EL), an individual learns that a previously learned behavior no longer fulfills its original purpose, or is no longer relevant. Recent studies have contradicted earlier theories that EL comprises forgetting, or the inhibition of the previously learned behavior, and indicate that EL comprises new associative learning. This suggests that the hippocampus is involved in this process. Empirical evidence is lacking however. Here, we used fluorescence *in situ* hybridization of somatic immediate early gene (IEG) expression to scrutinize if the hippocampus processes EL. Rodents engaged in context-dependent EL and were also tested for renewal of (the original behavioral response to) a spatial appetitive task in a T-maze. Whereas distal and proximal CA1 subfields processed both EL and renewal, effects in the proximal CA1 were more robust consistent with a role of this subfield in processing context. The lower blade of the dentate gyrus (DG) and the proximal CA3 subfields were particularly involved in renewal. Responses in the distal and proximal CA3 subfields suggest that this hippocampal subregion may also contribute to the evaluation of the reward outcome. Taken together, our findings provide novel and direct evidence for the involvement of distinct hippocampal subfields in context-dependent EL and renewal.

## Introduction

A fundamental and indispensable ability of the brain comprises learning of new information and the creation of responses to it, through which stimulus-response associations are formed. Equally important is the brain’s ability to distinguish when a former learned response is no longer valid and, therefore, should no longer be implemented in a stimulus-response. One example of the above-mentioned phenomena is acquired appetitive/aversive behavior, that is followed by extinction learning. Extinction learning of conditioned appetitive/aversive behavior plays a key role in the ability of an individual to interact in a flexible way with the environment (Taylor et al., 2009).

Despite the fact that extinction learning has been studied from a behavioral point of view for almost a century, the precise physiological principles underlying this behavioral process remain unclear. Based on the classical studies of Rescorla and Wagner (1972), the extinction of previously acquired associations should lead to an erasure of the original memory trace. However, Pavlov (1927) found behavioral evidence of spontaneous recovery (i.e., renewal) of a former conditioned response, subsequent to extinction training, that is incompatible with the idea of an erasure of the original association. He suggested that extinction involves inhibition of the learned behavioral response. Subsequently, several researchers proposed that the molecular mechanisms underlying the acquisition and/or consolidation of extinction memory are similar to those described for the acquisition and/or consolidation of the original contextual learning (Lattal et al., 2003; Szapiro et al., 2003; Delamater and Lattal, 2014). According to several researchers, extinction may be understood as new learning involving new memory formation, in conjunction with the conservation of the original memory trace that is also associated with decreased responding in memory tasks (Bouton et al., 2006; Archbold et al., 2010). Interestingly, these two core concepts are not mutually exclusive: part of the original trace might be erased within brain regions, whereas other areas retain and update this information. In line with this possibility, and despite the successful occurrence of extinction learning, spontaneous recovery can occur if the individual is re-exposed, after a delay in time, to the context in which the original experience was learned (André and Manahan-Vaughan, 2015; André et al., 2015a; Packheiser et al., 2019). This renewal of the original learned behavior suggests that the original memory trace is at the very least, partly conserved during extinction: an interpretation that is supported by brain imaging studies in human subjects (Lissek et al., 2013).

The behavioral context plays a key role in extinction learning and renewal (Bouton, 2004). Here, discriminating between aversive and appetitive forms of extinction learning may help extricate the role of specific brain structures in this process. The vast majority of studies of the neural basis of the extinction learning to date, have addressed this phenomenon from the perspective of aversive conditioning (Szapiro et al., 2003; Vianna et al., 2003; Cammarota et al., 2007; Kim and Richardson, 2009; Ernst et al., 2017) and suggest that the brain areas that encode fear conditioning might be the same as those involved in aversive extinction learning (Akirav and Maroun, 2007; Vlachos et al., 2011). Given its essential role in spatial, associative and context-dependent learning (for a review see McDonald and Mott, 2017), the hippocampus seems a likely location for the encoding of context-dependent extinction learning. Indeed, a specific role for the hippocampus in the extinction of conditioned *aversive* experience has been proposed (Vianna et al., 2003; Bouton et al., 2006; Herry et al., 2010; Orsini et al., 2011; Cleren et al., 2013; Chang et al., 2015; Nagayoshi et al., 2017). To what extent the hippocampus contributes to extinction learning of *appetitive* experience is less clear, although this seems likely (Conejo et al., 2013; Méndez-Couz et al., 2014, 2015b). Correlative evidence has been provided by studies that reported that blockade of neurotransmitter receptors, known to be important for hippocampal synaptic plasticity and hippocampus-dependent associative learning, also prevent context-dependent extinction learning and are involved in its reinstatement (André and Manahan-Vaughan, 2015; André et al., 2015b). Recent studies indicate that the hippocampus functionally differentiates between temporal, spatial and non-spatial experience, by means of robust proximodistal segregation of encoding of this information in CA1 and CA3 subfields, as well as the upper and lower blades of the dentate gyrus (DG; Beer et al., 2014, 2018; Hoang et al., 2018). Although the DG seems to provide an instructive signal that supports hippocampal encoding of memories, its role during extinction and retrieval is controversial and poorly understood (Méndez-Couz et al., 2015a, 2016; Bernier et al., 2017). All these findings suggest that the hippocampus may be able to differentiate between different components of an appetitive experience, including extinction learning and renewal.

In the present study, we examined to what extent the hippocampus is involved in extinction learning and (behavioral) renewal of an appetitive spatial memory task conducted in a T-maze. Using fluorescence *in situ* hybridization of somatic immediate early gene (IEG) expression, we observed a regional, temporal and functional differentiation of the contribution of hippocampal subfields in extinction-learning or renewal of the learned behavior. The encoding of both experiences by the same neurons was detected in specific subfields. Our findings suggest that extinction learning of a spatial appetitive task comprises an update of the previously learned experience, rather than *de novo* learning of the adapted behavior and provide novel and direct evidence for subfield-specific hippocampal involvement in context-dependent extinction learning and renewal of a context-dependent spatial appetitive task.

## Materials and Methods

The study was carried out in accordance with the European Communities Council Directive of September 22nd, 2010 (2010/63/EU) for care of laboratory animals and all experiments were conducted according to the guidelines of the German Animal Protection Law. They were approved in advance by the North Rhine-Westphalia (NRW) State Authority (Landesamt für Arbeitsschutz, Naturschutz, Umweltschutz und Verbraucherschutz, NRW). All efforts were made to reduce the number of animals used.

### Animals

Male Wistar male rats (280–330 g) were used in this study. Prior to any other manipulation, the animals were handled for 5 days by the experimenter. They were weighed prior to commencing the study and maintained at 85% of their initial body weight. They had *ad libitum* access to water. Animals were housed in sibling groups in temperature and humidity-controlled purpose-designed animal housing containers in a quiet room with a 12-h light-dark cycle.

### Behavioral Apparatus

**Figure 1:**
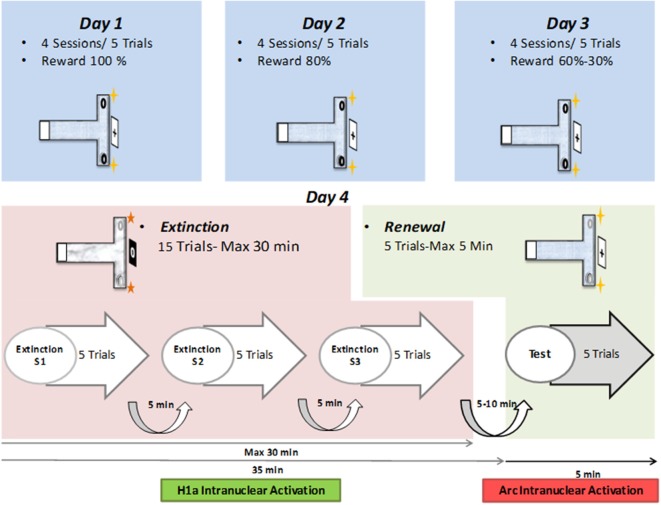
Layout of behavioral protocol. Top: on day 1, in context “A”, animals participate in four sessions, comprising of five consecutive trials each (separated by 5-min intervals) that include a reward probability of 100%. On Day 2, four sessions of five trials at 80% probability is repeated. On Day 3, the reward probability of the first two sessions is 60%, and of the last two sessions is 30%. Middle: on day 4, 15 extinction learning trials (maximally 30 min in total duration) are followed by five renewal trials (for maximally 5 min duration). During extinction learning, the context (floor pattern, distal cues, local odor cues) is changed to context “B.” During the renewal trials, context “A” is restored. Bottom: timeline for the extinction learning and renewal trials. The 15 trials are split into three sessions of five trials each. Each session is followed by a pause of 5 min duration. Five to ten minutes after the conclusion of the extinction trials, the renewal trials are commenced.

Experiments were carried out in an elevated T-maze composed of a starting box (25 × 20 cm) with a sliding door that separated the starting box from the main corridor (100 × 20 cm). At the end of the corridor, two side arms (10 × 40 cm) were positioned in a perpendicular manner to the main corridor (Wiescholleck et al., 2014; Figure 1).

Two different contexts were used for the different phases of the experiment. Context “A” describes the conditions used for the acquisition and renewal trials, whereas context “B” described the conditions used in the extinction learning trials. For each context, the T-maze differed in terms of the floor pattern, the distal cues positioned outside the maze and a faint odor that was placed at the end of the side arms (Wiescholleck et al., 2014). The room was faintly illuminated during experiments and animal behavior was recorded by means of a monitoring system (Videomot; TSE Systems, Bad Homburg, Germany), to permit offline analysis.

During the experiment, a small number of chocolate sprinkles (Dr. Oetker, Germany) were placed in a floor indentation at the end of the target side-arm. These could not be seen from afar and served as the appetitive reward.

### Habituation

During habituation, a smooth plastic floor covering was used that was distinct from that used in Context A and B. No odor, or distal, spatial cues were present.

Animals familiarized themselves with the T-maze on the 2 days prior to commencing the study. On the first habituation day, rats were placed in the starting box (Figure 1), the door between the box and the maze was opened and they were allowed to freely explore the maze for 5 min. Chocolate sprinkles were scattered in both side-arms of the maze to motivate exploration behavior.

The second habituation day consisted of two sessions of two trials each, 5 min apart. The food reward was placed only in a small indentation in the floor at the end of each side-arms. Rats explored the maze until the reward was found, or a maximal time of 2 min had elapsed, after which the animals were guided back to the starting position.

### Acquisition Trials in Context A

Animals underwent 3 days of acquisition trials. Each day consisted of four sessions of five trials. Each trial consisted of a maximal time of 2 min. The trial was concluded if the animal found the found reward before 2 min had elapsed. Each trial had an inter-trial interval of 15 s. Each session was interleaved with a 5-min pause.

Rats left the starting box and explored the maze. A correct and an incorrect arm were predetermined and remained consistent for all trials. A food reward was placed in an indentation located at the end of the target (correct) side-arm. The reward could not be seen from afar: the animal had to approach the indentation to find it. If the animal chose to enter the wrong (non-rewarded) arm, the exit to the main corridor was blocked and the animal was contained in the non-rewarded arm for a period of 15 s before being allowed to exit the arm and return to the starting position.

If a rat failed to move during 30 s in a trial, the entrance door of the maze was closed and the animal was not allowed to participate in the trial. Such non-decision trials were excluded from the statistical analysis of right or wrong choice performance.

Rats participated in 20 trials in total per day over three contiguous days. The reward probability decreased from 100% in the first day to 80% on the second day, 60% in the first 10 trials of the third day and a final probability of 30% in the last 10 trials of the last day (Figure 1). We previously reported that the reward probability reduction from 100% to 30% on days 1–3 helps to augment the perseverance of the animals in engaging in the T-maze task (André et al., 2015a,b). Without this form of training, testing contextual changes during repeated extinction trials in the absence of a food reward would not have been possible. Animals that did not reach the learning criterion, of at least 80% of correct choices, by the final trial of day three, were excluded from the study.

### Extinction Learning in Context B and Renewal in Context A

On the day after the conclusion of the acquisition trials (i.e., on Day 4), animals participated in an extinction learning protocol (in context “B”) that was immediately followed by re-exposure to context “A” to trigger renewal (Figure 1).

The protocol consisted of three sessions of five trials during which the animals were released from the start box and could freely explore the maze until they entered an arm, or until maximally 2 min had been spent in the maze. The intersession time was 5 min and the entire extinction learning phase was 25 min in total. After the last extinction learning trial, animals rested for 10 min in their home cage before participating in the renewal trial. For this, the context was changed back to context “A”. Rats engaged in five trials, as described above, for a maximum of 5 min. The timing of these events was planned such that somatic Homer1a expression served as a biomarker for the encoding of the extinction learning event and the somatic expression of Arc indicated renewal (see *in situ* hybridization methods below).

During both extinction learning and renewal, no rewards were present in the T-maze at any time. Furthermore, the animals were not restrained in a side-arm because of a wrong decision.

### Cohort Descriptions

Before starting the study, animals were randomly divided into three cohorts, Experimental (EXP) animals participated in the appetitive T-maze protocol as described above. In addition, two control cohorts were included: Control “naïve” animals (N) participated in the T-maze protocol, but no appetitive rewards were offered at any stage of the protocol. By contrast, animals in the “control aleatory reward” (CAR) group received rewards that were randomly provided through all three elements of the protocol (acquisition, extinction learning and renewal).

### Tissue Preparation

Immediately after the final renewal trial had concluded on day four, brains were removed within a maximal time of 2 min, frozen rapidly in −40°C isopentane, covered with parafilm and aluminum foil and stored at −80°C. Coronal sections (20 μm) of the brain were cut at −20°C in a cryostat (Microm HM-505E, Heidelberg, Germany) and then mounted on gelatinized slides. In order to facilitate the later proper localization of regions of interest, every 12th coronal section underwent Nissl staining. Regions of interest were subsequently verified using the stereotaxic atlas of Paxinos and Watson (2004).

### *In situ* Hybridization

The goal of this procedure was to examine the somatic expression of the IEGs, Homer1a and Arc. Due to the brief period of transcription (<10 min) of these genes and the difference in sizes of their primary transcripts, the somatic expression for Homer1a occurs 25–30 min after a novel experience and the somatic expression of Arc occurs <10 min after a novel experience (Guzowski et al., 1999; Vazdarjanova et al., 2002). Thus, in the present study Homer1a was used as a biomarker to identify hippocampal neurons that participated in extinction learning, whereas Arc expression indicated hippocampal neurons that participated in renewal.

Brain sections were treated using a previously established double fluorescence *in situ* hybridization protocol to reveal Homer 1a and Arc expression as already described (Grüter et al., 2015; Hoang et al., 2018). In short, tissue sections were fixed and acetylated in paraformaldehyde [PFA, 4% (ice-cold) 10 min], washed in saline sodium citrate (SSC) twice, and placed for 10 min in acetic anhydride solution (96.96% diethyl pyro carbonate (DEPC)-water, 0.89% NaCl, 1.62% triethanolamine, 0.52% acetic anhydride). After an additional five washes with SSC, tissue sections were prehybridized in prehybridization buffer (1:1, SSC: prehybridization buffer) for 30 min at room temperature (RT) followed by an hybridization process (Grüter et al., 2015). For this purpose, 1 ng/μl of RNA probe in hybridization buffer was applied, comprising 20/1,000 μl of Homer1a-Biotin and 20/1,000 μl Arc-Digoxigenin (50:1:1) in hybridization buffer. The solution was kept at 90°C for 5 min then chilled on ice to prevent reannealing until addition onto each glass slide. The diluted probe was added and samples were incubated in the humidified hybridization chamber (56°C, overnight).

One day after the abovementioned procedure, tissue sections underwent stringency washings to remove non-specific and repetitive RNA hybridization. First steps comprised five rinsing steps in SSC at 56°C, followed by RNase A (50 μg/100 ml 2× SSC) at 37°C, followed by rinsing with diluted SSC for 10 min at 37°C and three washings with diluted SSC, from 37°C to 56°C, finally an additional two washings at RT and a Tris-buffered saline (TBS) rinse was conducted to bring back the p.H to 7.5.

For the signal detection of both Homer1a-Biotin, and Arc-DIG, streptavidin was used, so the signal detection had to be performed sequentially. For Homer1a-Biotin an additional blocking step with 1% bovine serum albumin (BSA) in TBS-Tween of 70 min was carried out in a humidity chamber before the first antibody, streptavidin CY2 (Dianova, Cat# 016-220-084, RRID:AB_2337246) was applied at 1:250, 1% BSA: TBS-Tween, 30 min. An enhancement step was included, in which sections were incubated with b-Anti-Streptavidin (Vector Laboratories Cat# BA-0500, RRID: AB_2336221) at 1:100 in 1% BSA in TBS-Tween, followed by TBS washings and a *de novo* incubation with Streptavidin CY2 in the same conditions at before. Sections were rinsed in TBS and preserved overnight at 4°C.

One day later, the somatic Arc signal was detected by Arc-Dig immunohistochemistry. In order to reduce unspecific background staining, endogenous peroxidase was blocked by 0.3% H_2_O_2_ and after that, endogenous biotin and electrostatic loading of proteins were reduced by 20% avidin (Vector Labs, Cat# SP2001). Afterwards, the primary antibody for Anti-Digoxigenin was applied at 1:400 (Roche, Cat #11207733910, RRID:AB_514500) in 1% BSA (Sigma Aldrich, St. Louis, MO, USA) in TBS-Tween 20% biotin (Avidin-Biotin Blocking Kit) for 90 min at RT. The sections were newly washed in TBS and a biotinylated Tyramid (bT)-enhancement step was performed for 20 min, consisting of 1% bT and 0.3% H_2_O_2_ in TBS. The second antibody was applied after new rinsing in TBS, Steptavidin Cy5 (Jackson ImmunoResearch Labs Cat# 016-170-084, RRID:AB_2337245) 1:2,000 in 1% BSA TBS-Tween. In order to label the nuclei of the cells, 4′,6-diamidino-2-phenylindole (DAPI, Invitrogen, Carlsberg, CA, USA) was added in a concentration of 1:10,000. Slides were finally rinsed in TBS and distilled water, air-dried in a photo-box and mounting with fluorescence specific medium (Dianova SCR-38447).

### Quantification

**Figure 2:**
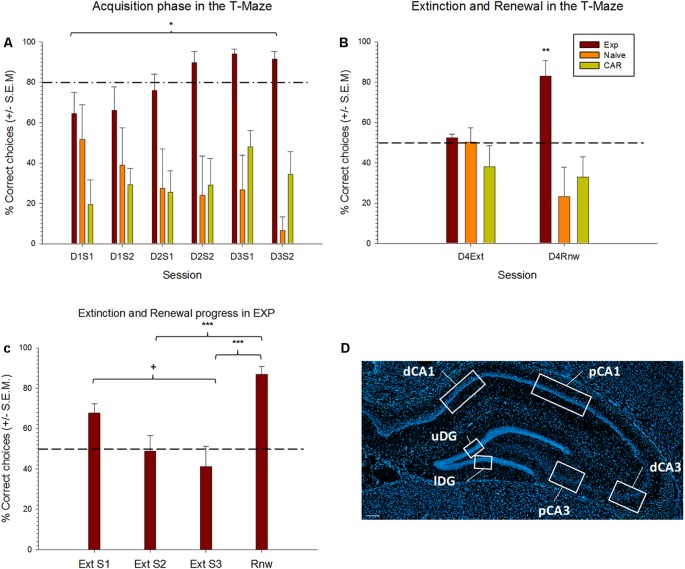
Extinction learning and renewal occurs in the test, but not control, animals. **(A,B)** Learning of a spatial appetitive task, extinction learning and renewal in three test groups. Experimental (EXP) animals learned that a specific side-arm of the T-Maze is rewarded. By the final trials of day 3, reward probability is 30%. No reward is given during extinction learning or renewal trials. Control “naïve” animals never receive a reward. Control aleatory reward (CAR) animals receive a reward with a 70% probability during all phases of the protocol. **(A)** For the acquisition trials, results from the first two sessions and last two sessions of each day are pooled into two groups (S1, S2) for each respective day. EXP animals successfully acquire the spatial learning task as demonstrated by the percentage of correct choices reaching above 80% (dashed line) by Day 2 (D2S2). CAR animals persist in search behavior that is at chance levels. Naïve animals show a decline in choice behavior by the final two trial blocks of day 3 (D3S2). **(B)** On Day 4, extinction learning (Ext) is tested in context “B.” All groups perform at chance levels (dashed line) consistent with extinction learning occurring in the EXP group (and random search behavior occurring in both control groups). EXP animals show significant renewal (Rnw) behavior. Control animals remain at chance levels. **(C)** Differences across extinction and renewal sessions in the experimental (EXP) group. EXP animals engaged in extinction learning, as demonstrated by the decreasing percentage of correct choices across extinction training sessions. Differences were found between first and last extinction sessions (^+^*p* = 0.004), reaching, by the second block, levels around the chance level, as indicated by a dashed line. Afterward, a renewal effect was evident when animals were confronted again with context A. Here, the percentage of correct responses was significantly higher than in the second and the third sessions of extinction learning (*p* ≤ 0.001). **(D)** DAPI stained section of the dorsal hippocampus showing the regions of interest scrutinized in the distal and proximal CA1, distal and proximal CA3 and upper and lower blades of the Dentate Gyrus (DG). Scale bar: 200 μm. *p < 0.05, **p < 0.01, ***p < 0.001.

For the *in situ* hybridization, we analyzed representative and randomly chosen small areas within the regions of interest of the CA1, CA3 and DG of the dorsal hippocampus measured at −3.30 mm from Bregma (Figure 2C). In addition, Nissl staining using 1% toluidine blue was performed for surveillance of tissue quality and spatial orientation. Furthermore, negative controls were prepared for supervision of specificity. For this purpose, the probe was omitted in those slides that then underwent the abovementioned staining protocols. No intranuclear staining could be observed in these negative controls, indicating that the staining observed in the test slides was specific. Images were acquired using a LEICA (Nussloch, Germany) confocal microscope. Z stacks of 0.5 μm thickness were acquired with a 60× oil lens.

Only putative cornus ammonis pyramidal and dentate gyrus granule cells were included in the analyses. Putative glial-cell nuclei were identified and discarded based on their smaller nuclear size, and bright, uniform nuclear DAPI counterstaining (Guzowski and Worley, 2001). These cells do not express Arc and Homer1a (Vazdarjanova et al., 2002), consistent with the idea of these oncogenes being expressed mostly in excitatory neurons (Cirelli and Tononi, 2000).

Z- stacks were analyzed using Fiji ImageJ image software program (Rueden et al., 2017) and positive cell results were manually counted and expressed as percentage of the total neuronal nuclei analyzed per subfield (proximal and distal parts of CA1, CA3 and upper and Lower blades of DG; Figure 2C) and animal. To prevent bias, the experimenter was unaware of the behavioral condition for each image analyzed.

### Statistical Analysis

In all cases through the experiment, a *p*-value ≤ 0.05 was considered as statistically significant. Data were analyzed using Sigmastat 11 (Systat Software Inc., Chicago, IL, USA).

For behavioral data, a two-way analysis of variance (ANOVA) mixed-model with repeated measures was applied to evaluate possible group differences in the number of correct choices (correct entries in the rewarded arm) across training sessions.

*Post hoc* test (Tukey HSD tests) were used to further analyze group differences in case of significant interaction between group and training sessions. They were also used to evaluate differences across training sessions in each experimental group. During the acquisition trials, the animals participated in four sessions of five trials each. For the analysis of acquisition behavior, daily trials were divided into two sessions of 10 trials each, and averages were calculated. For example, in Figure 2A, D1S1 describes the outcome of the first two sessions on day 1, D1S2 describes the final two sessions of day 1. The same principle was followed for days 2 and 3. Acquisition behavior was then compared across the experimental (EXP), control naive (N) and CAR (CAR) cohorts. A separate analysis was conducted across the three cohorts for their behavior during extinction learning or renewal.

A one-way repeated measures ANOVA was used to evaluate the differences in the percentage of correct choices across extinction sessions and the renewal session in the Experimental group. The Holm–Sidak method was used as a *post hoc* analysis to isolate the differences across sessions.

A two-way mixed-model ANOVA with repeated measures was carried out to determine the level of exploration of the animals. Here, the percentage of trials in which the animals left the start box and entered into the maze was analyzed per day and session in the same fashion as described above. *Post hoc* test (Tukey HSD tests) were used as for pairwise comparisons in case of significant differences between groups or training sessions.

For *in situ* hybridization data, the upper and lower blades of the DG, and proximal-distal parts of the CA1 and the CA3 were averaged per animal and sampled region. Differences in somatic expression of Arc and Homer1A were then assessed using a one-way ANOVA of a respective region of interest. The Kruskal–Wallis test was used as a nonparametric method in cases were the equal of variances test failed. Either the Holm–Sidak method, or the Dunn’s method were used as *post hoc* multiple comparison tests.

## Results

### Acquisition Successfully Occurs in Animals That Participate in the Appetitive Learning Task, but Control Animals Fail to Learn

Animals first participated in 3 days of acquisition trials in context “A” (Figure 1). During this time, for the test (EXP) animals (*n* = 8), the target arm was rewarded to a probability level of 100% on the first day, 80% on the second day, 60% during the first 10 trials of the third day and 30% in the last 10 trials of the third day (Figure 1). The naïve (N) cohort (*n* = 8) never received a reward, and the control aleatory reward (CAR) animals (*n* = 8) received a reward during all sessions that was given with a 70% probability in a pseudorandom manner. During the 3 days of acquisition, the experimental animals successfully learned the task (Figure 2A). The RM ANOVA test revealed a statistically significant interaction between group and session *F*_(14,146)_ = 1.98, *p* = 0, 023. Specifically, there was a main effect of group *F*_(2,146)_ = 19.22, *p* < 0, 001) that depends on the specific session. On day three, nine out of 10 trials were performed correctly, reaching the learning criterion of at least 80% of correct choices. By contrast, no significant difference in behavioral performance was found between the first and the last sessions for the two control groups (Tukey test for N (*p* = 0.63) and for CAR (*p* = 0.96) groups), that maintained their performance around chance levels (Figure 2A).

Specifically, a significant difference between EXP and the CAR cohorts became apparent from the last session of the first day (Tukey test, *p* = 0.02) until the last session of the third day (*p* ≤ 0.01). A different between EXP and N cohorts was evident from the second session on the first day (*p* ≤ 0.05) until the third day (*p* = 0.01). No significant difference was evident between the N and CAR cohorts through the entirety of the acquisition trials (Figure 2A).

### Extinction Learning Occurs in Test, but Not Control Animals

During extinction learning, animals were exposed to context “B” (Figure 1). When correct choice behavior during the final trials of day 3 (acquisition) and the trials on day 4 (extinction learning) was compared, a significant decline in the correct choice behavior was evident in the experimental (EXP) group (Tukey test, *p* ≤ 0.05). This effect is consistent with the occurrence of extinction learning. No significant difference in choice behavior was found between days 3 and 4 in the N or CAR cohorts, maintaining the chance level of their performance (Tukey test, *p* = 0.14 and *p* = 0.99, respectively). Furthermore, no significant differences were found between all three cohorts in the extinction phase: all groups made 50% correct choices on average (Figure 2B).

When the extinction and renewal progression were analyzed in the experimental groups across learning sessions, significant differences between sessions were found [*F*_(7,21)_ = 12.41, (*p* ≤ 0.001)]. *Post hoc* comparisons using the Holm–Sidak method indicated that the mean score for the first extinction session was significantly different from the last extinction session (*p* = 0.004).

### Renewal of the Previously Learned Behavior Occurs Only in the Experimental Cohort

To examine if renewal of the previously learned behavior occurred, the animals were re-exposed to context “A” after the conclusion of the extinction learning trials. Specifically, a significant increase in correct choice behavior was evident in the EXP cohort when the renewal trials were compared with the extinction phase (*Post hoc* Tukey test, *p* ≤ 0.05). The number of correct choices in the renewal phase was not significantly different from the last trials of the acquisition phase (*p* = 0.99).

By means of an independent analysis of the Extinction sessions, we found that the renewal session significantly differed both from the second extinction session (Holm–Sidak, *p* ≤ 0.001), as well as from the third extinction session (*p* ≤ 0.001).

A significant difference was also evident in the choice behavior of the EXP cohort compared to both N and CAR cohorts (*post hoc* Tukey’s test, *p* ≤ 0.01 in both cases). A comparison of the control groups revealed no significant difference in their performance (*p* = 0.69).

To examine the exploratory behavior of the animals between groups, we analyzed the number of times animals left the starting box and entered into the maze per session. The two-way RM ANOVA showed an effect of group (*F*_(2,147)_ = 1.91, *p* ≤ 0.001) and an effect of session (*F*_(7,147)_ = 1.19, *p* = 0.039). There was no interaction however between group and session (*F*_(14,147)_ = 1.198 *p* = 0.28). The *post hoc* Holm–Sidak tests revealed differences in the number of times animal entered into the maze at the end of the acquisition between experimental animals and the N group (*p* ≤ 0.01) and between the rewarded CAR and N animals (*p* = 0.01), which never received a reward in the maze.

### The Same Cell Assemblies of the CA1 Region Are Active During Both Extinction Learning and Renewal

We assessed somatic IEG expression resulting from extinction learning and renewal in the same animal by exploiting the fact that Homer1a and Arc expression are triggered 25–30 min, and <10 min, respectively after an experience or learning event (Guzowski et al., 1999; Guzowski and Worley, 2001; Vazdarjanova et al., 2002; Nalloor et al., 2012). Thus, we used Homer1a as a biomarker for the hippocampal encoding of extinction learning and Arc as a biomarker for the renewal event. We subdivided the hippocampus into regions of interest that allowed scrutiny of the distal and proximal CA1 regions, the distal and proximal CA3 regions, and the upper and lower blades of the DG (Figure 2C), based on recent findings that subcomponents of spatial information are functionally discriminated by these subfields (Hoang et al., 2018).

**Figure 3:**
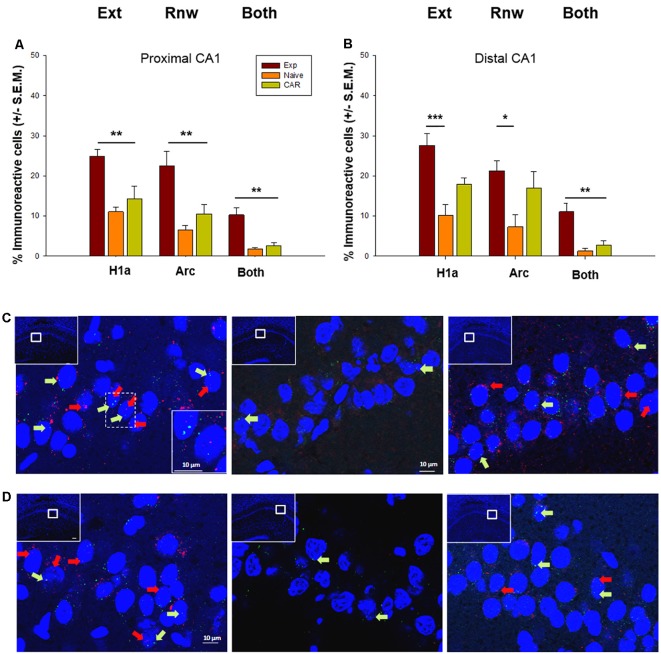
Immediate early gene (IEG) expression in the CA1 region following extinction learning and renewal. **(A,B)** Significant increases in the percentage of Homer1a (H1a)-positive cell nuclei are evident in the proximal **(A)** and distal **(B)** CA1 regions after extinction learning (Ext) in experimental (EXP) animals compared to “naïve” (never rewarded) animals, and animals that received a control aleatory reward (CAR). Arc expression is also increased in both subfields after the renewal trials. Both proximal **(A)** and distal **(B)** subfields show double-labeled nuclei (“Both”) that indicate IEG activation triggered by both extinction learning and renewal. **(C,D)** Photomicrographs show Arc mRNA expression (red dots, indicated by arrows) and H1a (green dots and arrows) in the proximal CA1 **(C)** and distal CA1 **(D)** regions of Experimental (EXP) control naïve animals (N) or CAR animals. Blue: nuclear staining with DAPI. The upper left rectangles in each microphotograph include photomicrographs of the complete section from which the sampling sites were taken. Images were taken using a 63× objective, or a 5× for the complete field view. Scale bar: 10 μm. *p < 0.05, **p < 0.01, ***p < 0.001.

With regard to the CA1 region, we detected significant increases in somatic Homer1a and Arc expression as a result of extinction learning and renewal in the EXP group (*n* = 7; Figure 3), compared to expression detected in the control N (*n* = 7) and CAR cohorts (*n* = 7). Effects were apparent in both the proximal and distal subregions of the hippocampus: In the proximal CA1, ANOVA revealed differences between groups in the number of Homer1a positive cells (*F*_(2,16)_ = 12.87, *p* ≤ 0.01) that was confirmed by a* post hoc* test (Holm–Sidak, *p* ≤ 0.05). Significant differences in the number of Arc positive cells were also identified between groups (ANOVA: *F*_(2,16)_ = 11.76, *p* ≤0.01, Holm–Sidak, *p* ≤ 0.05). In the distal CA1, differences were found in the number of Homer1a cells using ANOVA (*F*_(2,17)_ = 9.6, *p* = 0.01), and *post hoc* tests indicated that specific differences occurred between the Exp and N groups (*p* < 0.01). In addition, significant differences were found in the number of Arc reactive cells (ANOVA: *F*_(2,18)_ = 3.73, *p* = 0.04), whereas *post hoc* tests (Holm–Sidak, *p* ≤ 0.05) indicated specific differences between the Exp and N groups (*p* = 0.01).

Strikingly, a significant elevation in the number of double-labeled cells was detected in the EXP cohort compared to the N and CAR groups, both in the distal CA1 (ANOVA: *F*_(2,17)_ = 20.49, *p* < 0.01, Holm–Sidak, *p* ≤ 0.05) and in the proximal CA1 (Dunn’s Method, *p* ≤ 0.01). This suggests that extinction learning may serve to update an established representation that is processed in the CA1 region (Figure 3).

### The Proximal CA3 Region Encodes Both Extinction Learning and Renewal

**Figure 4:**
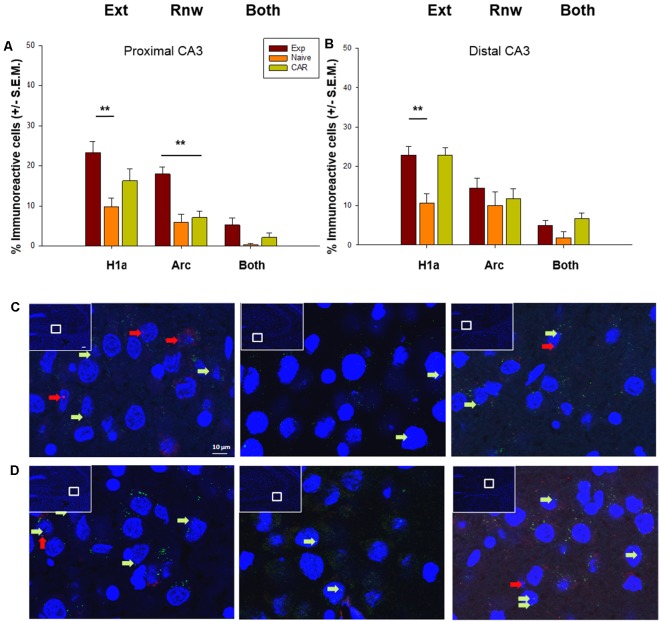
IEG expression in the CA3 region following extinction learning and renewal. **(A)** Significant increases in the percentage of Homer1a (H1a)-positive cell nuclei are evident in the proximal CA3 region after extinction learning (Ext) in experimental (EXP) animals compared to reinforcement-naïve animals. Arc expression also is increased in EXP animals after the renewal trials, compared to naïve (never rewarded) and animals that received a control aleatory reward (CAR). Double-labeled nuclei (“Both”) are not significantly different across groups. **(B)** Homer1a (H1a) expression is increased in EXP animals compared to naïve, but not CAR groups. Arc expression and double-labeled nuclei (“Both”) are not significantly different across groups. **(C,D)** Photomicrographs show Arc mRNA expression (red dots, indicated by arrows) and H1a (green dots and arrows) in the proximal CA3 **(C)** and distal CA3 **(D)** regions of Experimental (EXP) control naïve animals (N) or animals that had an aleatory reward (CAR). Blue: nuclear staining with DAPI. The upper left rectangles in each microphotograph include photomicrographs of the complete section from which the sampling sites were taken. Images were taken using a 63× objective, or a 5× for the complete field view. Scale bar: 10 μm. **p < 0.01.

When we scrutinized IEG expression in the soma of the proximal CA3 region we found that extinction learning triggered a significant elevation of Homer1a expression in the EXP group (*n* = 7) compared to naïve (N) controls (*n* = 7; *F*_(2,17)_ = 8.31, *p* ≤ 0.01; Figure 4), but effects were not significant when the expression of Homer1a was compared in the EXP and CAR (*n* = 7) groups (Holm–Sidak, *p* = 0.057; Figure 4). The expression of the H1a in the CAR group was also not statistically significant from the N group (Holm–Sidak, *p* = 0.087). Although a tendency towards an increase in double-labeled cells was evident, this was not significant. In the proximal CA3 region, Arc expression was elevated compared to N and CAR groups, consistent with a role for the CA3 region in renewal (ANOVA: *F*_(2,17)_ = 17.26; *p* ≤ 0.01, Holm–Sidak: *p* ≤ 0.01; Figure 4).

In the distal CA3 region, Homer1a expression was elevated in the Exp group compared to Naïve (N) controls (ANOVA: *F*_(2,17)_ = 13.70, *p* ≤0.01, Holm–Sidak, *p* ≤ 0.01). However, no differences were found in expression levels between the Exp and CAR groups (*p* = 0.88). By contrast, Homer1a expression was significantly different in CAR and N groups (Holm–Sidak, *p* ≤ 0.01). Taken together with observations made for the proximal CA3 this suggests that, in the CA3 region, the elevation in somatic Homer1a expression may have less to do with extinction learning *per se*, and more to do with the change in context and the anticipation of a change in reward conditions.

In the distal CA3 region, no change in Arc expression occurred in the Exp group compared to both control groups (ANOVA: *F*_(2,17)_ = 1.07 *p* = 0.36), indicating that the distal CA3 region does not encode renewal, or indeed engage in reactivation of the previously learned experience (Figure 4).

### The Dentate Gyrus Processes Renewal and Information Updating

Scrutiny of IEG expression in the DG revealed significant double-labeling of cells in the upper blade (H_2_ = 7.10, *p* = 0.03, *n* = 7 for all cohorts). This effect was evident even though Homer1a expression was equivalent in all three cohorts (ANOVA: *F*_(2,16)_ = 0.06 *p* = 0.94), and a tendency towards an increase in Arc expression that proved to be non-significant (H_2_ = 5.28, *p* = 0.07). This finding suggests that although only a low number of cells respond to novel, or information updating experiences in the DG (Hoang et al., 2018), the upper blade may be involved in the updating of the context-dependent experience.

**Figure 5:**
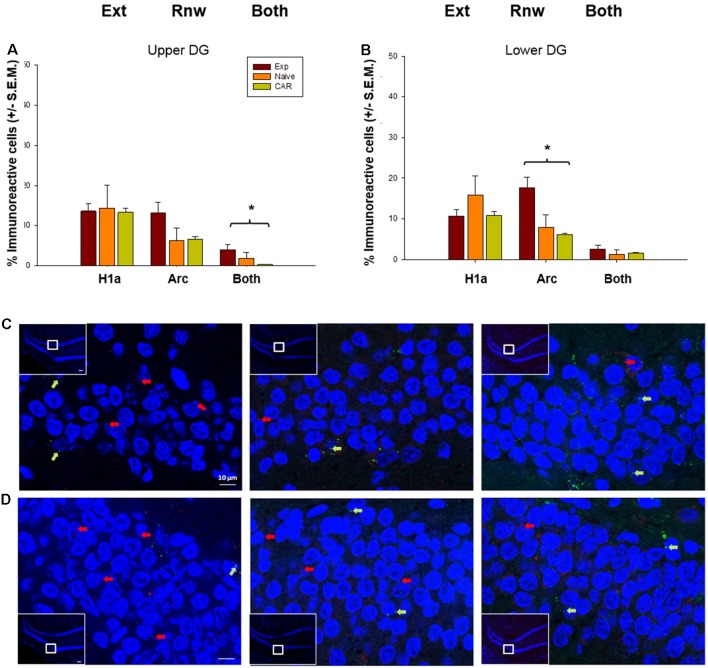
IEG expression in the DG following extinction learning and renewal. **(A)** After extinction learning (H1a) and renewal (Arc), IEG expression in the upper blade of the DG (Upper DG) is not significantly different across groups. Nonetheless, the number of double-labeled cells (“Both”) that show IEG activation triggered by both extinction learning and renewal is significantly increased in EXP animals compared to naïve (never rewarded) and animals that received a control aleatory reward (CAR). **(B)** In the lower blade of the DG (Lower DG), Arc IEG expression corresponding to renewal is elevated in EXP animals compared to naïve and CAR animals. No changes in Homer1a occur following extinction learning. Double-labeled nuclei (“Both”) are not significantly different across groups. **(C,D)** Photomicrographs show Arc mRNA expression (red dots, indicated by arrows) and H1a (green dots and arrows) in the upper blade of the DG **(C)** and lower blade of the DG **(D)** regions of Experimental (EXP) control naïve animals (N), or animals that received a CAR. Blue: nuclear staining with DAPI. The upper left rectangles in each microphotograph include photomicrographs of the complete section from which the sampling sites were taken. Images were taken using a 63× objective, or a 5× for the complete field view. Scale bar: 10 μm. *p < 0.05.

By contrast, the lower blade of the DG appears to be exclusively involved in renewal (Figure 4). Here, we observed a significant increase in Arc expression compared to the control cohorts (H_2_ = 8.43, *p* = 0.01, *post hoc* Dunn’s method, *p* < 0.05). By contrast Homer1a expression (ANOVA: *F*_(2,16)_ = 2.86, *p* = 0.09) and double-labeled cells (H_2_ = 2.97, *p* = 0.23) were equivalent across all groups (Figure 5).

## Discussion

In this study, we demonstrate for the first time that the hippocampus is intrinsically involved in the processing of both context-dependent extinction learning and renewal of a spatial appetitive experience of information processing. Furthermore, functional compartmentalization of hippocampal encoding of these experiences takes place.

We chose to include two control groups to exclude the possibility that motivation, or lack of it, would affect our results. In the “naïve” (N) control group (never rewarded), animals participated in all elements of the T-maze protocol without ever receiving a reward. In the CAR group (randomly rewarded), animals received a random reward with a probability of 70%, regardless of which stage of the protocol in which they were engaged. Our experimental (EXP) animals only received a reward during the acquisition trials (with a probability of 30% by the last trial of day 3).

We observed that during the acquisition trials, “naïve” animals that had never experienced reinforcement learning in the T-maze, participated in a lower number of trials, as compared to the experimental (test) and CAR animals. They also demonstrated a reduction in the number of decision trials. This could be linked to the reduced exploratory behavior associated to an absence of a reward in the maze, and an already familiar context. In contrast, the CAR animals showed a consistent number of pseudo-correct arm choices throughout the acquisition, extinction and learning phase, serving as a control for the presence of reward in the maze, and as a locomotion control.

After the acquisition phase, EXP animals successfully engaged in extinction learning of the previously reinforced behavior, thereby achieving a chance level of preference for the former goal arm. This reduced preference was significantly different from the last acquisition session of the experimental animals. However, no difference in performance was found during the extinction phase between groups, which is easily explained if we take into account that control animals have not acquired any arm preference, and therefore, maintained a random choice level in this phase.

Likewise, EXP animals exhibited renewal of the previously learned behavior in context “A.” Upon comparing the performance of the renewal session with the last acquisition session, it was observed that animals made a lower level of correct choices during renewal, but still performed better than during the extinction learning session. This pattern of behavior has been described for operant conditioning (Bouton, 2004; Bouton et al., 2006; Todd et al., 2014) and in the same T-maze paradigm used here, where extinction learning and renewal were separated by at least 24 h (André et al., 2015a,b). Our results furthermore demonstrate that the renewal effect also occurs even when the animal is confronted with the original context soon after extinction learning. Renewal, shortly after extinction learning and in the absence of a prolonged consolidation period, has also been demonstrated in pigeons (Packheiser et al., 2019). These results agree with those obtained for extinction learning of different kinds of reference memory tasks (Méndez-Couz et al., 2014, 2016) and concur with results in human studies, where during extinction in a novel context, participants who showed a renewal effect, had previously shown quicker extinction learning and increased hippocampus activation (Lissek et al., 2013; Chang et al., 2015).

We scrutinized the IEGs Homer1a and Arc that demonstrate a precise time-locked somatic expression following a behavioral experience. Whereas Homer1a achieves peak somatic expression 25–30 min after an experience, peak Arc expression occurs within 5–6 min of neuronal activation (Guzowski et al., 1999). Both IEGs rapidly disperse into the cytoplasm after somatic expression (Guzowski et al., 1999; Guzowski and Worley, 2001), Due to the brief period of transcription of these genes and the difference in sizes of their primary transcripts, somatic expression gives a very precise read-put as to the neurons that engage in encoding or processing of the experience (Nalloor et al., 2012). To determine whether extinction learning or renewal triggers *de novo* gene expression in the hippocampus, we conducted fluorescence *in situ* hybridization of Homer1a and Arc, whereby Homer1a served as a biomarker for extinction learning and Arc indicated the somatic location of renewal.

The EXP animals exhibited a relatively fast extinction response that became evident by the second session of extinction learning trials (Figure 2C). This is consistent with the effect of the context change on extinction learning in the T-maze paradigm used in the present study (André et al., 2015a,b). This contrasts with the much slower extinction response that occurs in this paradigm in the absence of a context change (André and Manahan-Vaughan, 2015; André et al., 2015a,b). Thus, the context change in the present study may have had an impact on somatic IEG expression. However, the timing of our experiments meant that the Homer1a “read-out” for extinction learning corresponded to the animals’ behavior in the second and third extinction learning trials (see Figures 1, 2C). Thus, although we cannot entirely exclude that the context change during extinction learning triggered initial neuronal encoding in its own right [as reflected by the higher-than-chance choice performance in the first extinction session (Figure 2C)], extinction learning should have been the primary determinant of Homer1a expression at the time-point of analysis.

We segregated our hippocampal regions of interest on the basis of past recent reports of functional discrimination by distinct hippocampal sub-compartments of temporal, non-spatial and spatial components of a behavioral experience (Beer et al., 2014; Hoang et al., 2018). In comparison to both control groups, EXP animals revealed a distinct and functionally differentiated expression of somatic IEGs as a consequence of extinction learning and renewal. We observed that the distal and proximal CA1 regions both engage in the encoding of extinction learning and in the renewal response. The effects were more pronounced in the proximal CA1 region. Somata that expressed both IEGs were also evident, suggesting that within the CA1 region extinction learning and renewal are encoded within the same neuronal network.

Neuroanatomically speaking, the distal CA1 and proximal CA3 regions may process information from the “what” visual stream, whereas the proximal CA1 and distal CA3 regions process information from the “where” visual stream (Amaral and Witter, 1989) in the context of the “two streams” hypothesis of visual information processing (Mishkin et al., 1983). Functional confirmation of this possibility has been provided in recent years (Chawla et al., 2005; Beer et al., 2014; Hoang et al., 2018). “What” information relates, for example, to item location in space, whereas “where” information characterizes the context in which the experience is made (Hoang et al., 2018). Our finding, that both the distal and proximal CA1 regions process extinction learning and renewal, suggests that appetitive spatial learning in the T-Maze incorporates knowledge about the context in which the animal finds itself (distal CA1 encoding), as well as the items (e.g., reward, or arm location) located in that space (proximal CA1 encoding). The pronounced response of the proximal CA1 region is consistent with the saliency of the spatial context on both the “A” and “B” spatial learning conditions used in our study. Motivation may play a greater role in experience encoding in the distal CA1 region. Here, IEG effects were only significant when the experimental group was compared with the control naïve (N) animal group. T-Maze exploration in the CAR group was encouraged by pseudorandom baiting of T- maze arms. This had an impact on IEG expression in the distal CA1 region both in the extinction learning and renewal conditions.

The finding that double-labeled cells were detected in both CA1 subfields are also in line with a putative role for the CA1 region in pattern completion (Mizumori et al., 1989; Hunsaker and Kesner, 2013; Kyle et al., 2015) and suggests that the extinction learning event may be encoded in the CA1 region the form of an update of the original representation (that is putatively re-activated during renewal). Alternatively, the renewal event serves to modify the extinction learning representation. This latter possibility cannot be excluded, given that in the present study the renewal event followed soon after extinction learning and was not punctuated by a consolidation phase.

In line with the likelihood that the change of context was a salient aspect of extinction learning and renewal, we found significant increases of both Homer1a and Arc in the proximal CA3 region (part of the “where” stream’). Although there was a tendency towards elevations of double-labeling of soma, effects were not significant. This suggests that in the CA3 region, extinction learning and renewal are processed by separate neuronal populations. This would align with a putative role for the CA3 region in context-dependent pattern separation (Knierim and Neunuebel, 2016; Loh et al., 2016; Sun et al., 2017) or in error correction (Knierim and Neunuebel, 2016). Others have shown a similar segregation of function within the CA3 region with regard to spatial information processing: the novel exploration of spatial cues in a defined context results in activation of somatic IEG expression in the proximal, but not distal, CA3 region (Hoang et al., 2018).

The elevations of Homer1a in the proximal CA3 region were significant in EXP animals compared to the non-reinforced naïve (N), but not compared to the pseudo-randomly rewarded (CAR) control group. No differences were found, however, between control groups. Although in the distal CA3 region, we also detected elevations of Homer1a in both EXP and CAR animals, we also found significant differences in between the rewarded EXP and N, as well as between CAR and N. This suggests that the elevations of Homer1a expression in the distal CA3 region were driven in EXP and CAR animals by an expectation of finding a reward, or the anticipation of a change in reward conditions, in contrast to the “naïve” animals that had never experienced a reward within the T-maze and did not show comparable IEG elevations. However, the EXP animals did not receive a reward in the extinction learning trial, whereas the CAR will have randomly received one. The elevation of somatic Homer1a expression following the change of the context in the extinction learning trials, may thus, reflect processing of a fulfilled, or failed, reward expectation. This interpretation aligns with recent studies that propose that the DG/CA3 circuit evaluates the outcome of an experience (Lee et al., 2017) and that co-activation of the DG/CA3 with the brain’s reward system, may underlie a reward-related enhancement of long-term memory (Loh et al., 2016).

Recently a functional segregation of spatial information processing has been demonstrated for the upper and lower blades of the DG (Hoang et al., 2018). Anatomically, the medial entorhinal cortex (part of the “where” stream) projects predominantly to the lower blade of the DG (Wyss, 1981; Tamamaki, 1997). We detected increased Arc expression in the lower blade consistent with the recruitment by renewal activation of context-dependent “where” information encoding. This would agree with the possibility that for context-dependent extinction learning a reactivation of the previous context is necessary, in line with the recruitment of hippocampal recall processes, for which the DG would be necessary (Bernier et al., 2017). Curiously, however, we also detected double-labeled soma in the upper blade of the DG, which predominantly receives “what” information *via* the lateral entorhinal cortex (Wyss, 1981; Amaral and Witter, 1989; Tamamaki, 1997). This may reflect the proposed role of the DG in enabling the precision of discrimination of spatial information (Baker et al., 2016; Hoang et al., 2018). Specifically, the upper blade has been proposed to be involved in the encoding of *distributed* directional cue information, as gradients of odors, or the shape of a polarized maze (Hoang et al., 2018). Our data are also consistent with previous findings showing that the DG is involved in the recall of an already known place (Emerich and Walsh, 1989; Méndez-Couz et al., 2015a).

## Conclusion

It is generally accepted that extinction learning does not comprise the destruction of the original memory trace (Lattal et al., 2003; Szapiro et al., 2003). However, it is still controversial which mechanisms subserve changes in the establishment of the original memory trace that is required for the extinction learning to effectively take place (Pagani and Merlo, 2019). Our results show increased activation of the distal and proximal CA1 regions during both extinction learning and renewal. Strikingly, an increased level of somata that express both H1a and Arc mRNA is also evident. This suggests that portions of the same neuronal ensembles may participate in both extinction learning and renewal within this hippocampal subfield and that the CA1 region may be involved in the encoding of multiple (“what” vs. “where”) facets of the extinction learning experience. By contrast, the CA3 region may support the encoding of context-related aspects of extinction learning and renewal within its proximal subfield, whereas the DG may support the discrimination of specific features of renewal and feature updating.

Taken together, our findings suggest that the extinction learning of robustly stored appetitive spatial behavior may occur in the form of an update of the previously learned representation, rather than a new learning process itself. The involvement of the “where” and what component pathways in this process suggests that encoding is highly context-dependent and visuospatial in nature. Furthermore, our results demonstrate that encoding and functional discrimination of distributed elements of context-dependent extinction learning of a spatial appetitive task occurs in the hippocampus.

## Data Availability Statement

The data that support the findings of this study are available from the corresponding author upon reasonable request.

## Ethics Statement

The animal study was reviewed and approved by Landesamt für Arbeitsschutz, Naturschutz, Umweltschutz und Verbraucherschutz, Nordrhein Westfalen, Germany.

## Author Contributions

The study was designed by DM-V and MM-C. Experiments were conducted by MM-C and JB and analyzed by all authors. MM-C and DM-V wrote the article.

## Conflict of Interest

The authors declare that the research was conducted in the absence of any commercial or financial relationships that could be construed as a potential conflict of interest.

## References

[B1] Akirav I. Maroun M. (2007). The role of the medial prefrontal cortex-amygdala circuit in stress effects on the extinction of fear. Neural Plast. 2007:30873. 10.1155/2007/30873 17502909 PMC1838961

[B2] Amaral D. G. Witter M. P. (1989). The three-dimensional organization of the hippocampal formation: a review of anatomical data. Neuroscience 31, 571–591. 10.1016/0306-4522(89)90424-7 2687721

[B4] André M. A. Gunturkun O. Manahan-Vaughan D. (2015a). The metabotropic glutamate receptor, mGlu5, is required for extinction learning that occurs in the absence of a context change. Hippocampus 25, 149–158. 10.1002/hipo.22359 25160592 PMC4322473

[B5] André M. A. Wolf O. T. Manahan-Vaughan D. (2015b). Beta-adrenergic receptors support attention to extinction learning that occurs in the absence, but not the presence, of a context change. Front. Behav. Neurosci. 9:125. 10.3389/fnbeh.2015.00125 26074793 PMC4444826

[B3] André M. A. Manahan-Vaughan D. (2015). Involvement of dopamine D1/D5 and D2 receptors in context-dependent extinction learning and memory reinstatement. Front. Behav. Neurosci. 9:372. 10.3389/fnbeh.2015.00372 26834599 PMC4720788

[B6] Archbold G. E. Bouton M. E. Nader K. (2010). Evidence for the persistence of contextual fear memories following immediate extinction. Eur. J. Neurosci. 31, 1303–1311. 10.1111/j.1460-9568.2010.07161.x 20345921

[B7] Baker S. Vieweg P. Gao F. Gilboa A. Wolbers T. Black S. E.. (2016). The human dentate gyrus plays a necessary role in discriminating new memories. Curr. Biol. 26, 2629–2634. 10.1016/j.cub.2016.07.081 27666968

[B8] Beer Z. Chwiesko C. Sauvage M. M. (2014). Processing of spatial and non-spatial information reveals functional homogeneity along the dorso-ventral axis of CA3, but not CA1. Neurobiol. Learn. Mem. 111, 56–64. 10.1016/j.nlm.2014.03.001 24657342

[B9] Beer Z. Vavra P. Atucha E. Rentzing K. Heinze H. J. Sauvage M. M. (2018). The memory for time and space differentially engages the proximal and distal parts of the hippocampal subfields CA1 and CA3. PLoS Biol. 16:e2006100. 10.1371/journal.pbio.2006100 30153249 PMC6136809

[B10] Bernier B. E. Lacagnina A. F. Ayoub A. Shue F. Zemelman B. V. Krasne F. B.. (2017). Dentate gyrus contributes to retrieval as well as encoding: evidence from context fear conditioning, recall and extinction. J. Neurosci. 37, 6359–6371. 10.1523/JNEUROSCI.3029-16.2017 28546308 PMC5490069

[B11] Bouton M. E. (2004). Context and behavioral processes in extinction. Learn. Mem. 11, 485–494. 10.1101/lm.78804 15466298

[B12] Bouton M. E. Westbrook R. F. Corcoran K. A. Maren S. (2006). Contextual and temporal modulation of extinction: behavioral and biological mechanisms. Biol. Psychiatry 60, 352–360. 10.1016/j.biopsych.2005.12.015 16616731

[B13] Cammarota M. Bevilaqua L. R. Vianna M. R. Medina J. H. Izquierdo I. (2007). The extinction of conditioned fear: structural and molecular basis and therapeutic use. Braz. J. Psychiatry 29, 80–85. 10.1590/s1516-44462007000100019 17435934

[B14] Chang D. I. Lissek S. Ernst T. M. Thurling M. Uengoer M. Tegenthoff M.. (2015). Cerebellar contribution to context processing in extinction learning and recall. Cerebellum 14, 670–676. 10.1007/s12311-015-0670-z 25863813

[B15] Chawla M. K. Guzowski J. F. Ramirez-Amaya V. Lipa P. Hoffman K. L. Marriott L. K.. (2005). Sparse, environmentally selective expression of Arc RNA in the upper blade of the rodent fascia dentata by brief spatial experience. Hippocampus 15, 579–586. 10.1002/hipo.20091 15920719

[B16] Cirelli C. Tononi G. (2000). Differential expression of plasticity-related genes in waking and sleep and their regulation by the noradrenergic system. J. Neurosci. 20, 9187–9194. 10.1523/jneurosci.20-24-09187.2000 11124996 PMC6773024

[B17] Cleren C. Tallarida I. Guiniec E. L. Janin F. Nachon O. Canini F.. (2013). Low-frequency stimulation of the ventral hippocampus facilitates extinction of contextual fear. Neurobiol. Learn. Mem. 101, 39–45. 10.1016/j.nlm.2012.12.017 23298787

[B18] Conejo N. M. Cimadevilla J. M. Gonzalez-Pardo H. Mendez-Couz M. Arias J. L. (2013). Hippocampal inactivation with TTX impairs long-term spatial memory retrieval and modifies brain metabolic activity. PLoS One 8:e64749. 10.1371/journal.pone.0064749 23724089 PMC3665627

[B19] Delamater A. R. Lattal K. M. (2014). The study of associative learning: mapping from psychological to neural levels of analysis. Neurobiol. Learn. Mem. 108, 1–4. 10.1016/j.nlm.2013.12.006 24333530 PMC4444052

[B20] Emerich D. F. Walsh T. J. (1989). Selective working memory impairments following intradentate injection of colchicine: attenuation of the behavioral but not the neuropathological effects by gangliosides GM1 and AGF2. Physiol. Behav. 45, 93–101. 10.1016/0031-9384(89)90170-4 2727146

[B21] Ernst T. M. Thurling M. Muller S. Kahl F. Maderwald S. Schlamann M.. (2017). Modulation of 7 T fMRI signal in the cerebellar cortex and nuclei during acquisition, extinction and reacquisition of conditioned eyeblink responses. Hum. Brain Mapp. 38, 3957–3974. 10.1002/hbm.23641 28474470 PMC6866767

[B22] Grüter T. Wiescholleck V. Dubovyk V. Aliane V. Manahan-Vaughan D. (2015). Altered neuronal excitability underlies impaired hippocampal function in an animal model of psychosis. Front. Behav. Neurosci. 9:117. 10.3389/fnbeh.2015.00117 26042007 PMC4438226

[B24] Guzowski J. F. McNaughton B. L. Barnes C. A. Worley P. F. (1999). Environment-specific expression of the immediate-early gene Arc in hippocampal neuronal ensembles. Nat. Neurosci. 2, 1120–1124. 10.1038/16046 10570490

[B23] Guzowski J. F. Worley P. F. (2001). Cellular compartment analysis of temporal activity by fluorescence *in situ* hybridization (catFISH). Curr. Protoc. Neurosci. 15, 1.8.1–1.8.16. 10.1002/0471142301.ns0108s15 18428454

[B25] Herry C. Ferraguti F. Singewald N. Letzkus J. J. Ehrlich I. Luthi A. (2010). Neuronal circuits of fear extinction. Eur. J. Neurosci. 31, 599–612. 10.1111/j.1460-9568.2010.07101.x 20384807

[B26] Hoang T. H. Aliane V. Manahan-Vaughan D. (2018). Novel encoding and updating of positional, or directional, spatial cues are processed by distinct hippocampal subfields: evidence for parallel information processing and the “what” stream. Hippocampus 28, 315–326. 10.1002/hipo.22833 29394518 PMC5947642

[B27] Hunsaker M. R. Kesner R. P. (2013). The operation of pattern separation and pattern completion processes associated with different attributes or domains of memory. Neurosci. Biobehav. Rev. 37, 36–58. 10.1016/j.neubiorev.2012.09.014 23043857

[B28] Kim J. H. Richardson R. (2009). Expression of renewal is dependent on the extinction-test interval rather than the acquisition-extinction interval. Behav. Neurosci. 123, 641–649. 10.1037/a0015237 19485571

[B29] Knierim J. J. Neunuebel J. P. (2016). Tracking the flow of hippocampal computation: pattern separation, pattern completion and attractor dynamics. Neurobiol. Learn. Mem. 129, 38–49. 10.1016/j.nlm.2015.10.008 26514299 PMC4792674

[B30] Kyle C. T. Stokes J. D. Lieberman J. S. Hassan A. S. Ekstrom A. D. (2015). Successful retrieval of competing spatial environments in humans involves hippocampal pattern separation mechanisms. Elife 4:e10499. 10.7554/elife.10499 26613414 PMC4733045

[B31] Lattal K. M. Mullen M. T. Abel T. (2003). Extinction, renewal, and spontaneous recovery of a spatial preference in the water maze. Behav. Neurosci. 117, 1017–1028. 10.1037/0735-7044.117.5.1017 14570551

[B32] Lee S. H. Huh N. Lee J. W. Ghim J. W. Lee I. Jung M. W. (2017). Neural signals related to outcome evaluation are stronger in CA1 than CA3. Front. Neural Circuits 11:40. 10.3389/fncir.2017.00040 28638322 PMC5461339

[B33] Lissek S. Glaubitz B. Uengoer M. Tegenthoff M. (2013). Hippocampal activation during extinction learning predicts occurrence of the renewal effect in extinction recall. Neuroimage 81, 131–143. 10.1016/j.neuroimage.2013.05.025 23684875

[B34] Loh E. Kumaran D. Koster R. Berron D. Dolan R. Duzel E. (2016). Context-specific activation of hippocampus and SN/VTA by reward is related to enhanced long-term memory for embedded objects. Neurobiol. Learn. Mem. 134, 65–77. 10.1016/j.nlm.2015.11.018 26708279 PMC5045461

[B35] McDonald A. J. Mott D. D. (2017). Functional neuroanatomy of amygdalohippocampal interconnections and their role in learning and memory. J. Neurosci. Res. 95, 797–820. 10.1002/jnr.23709 26876924 PMC5094901

[B36] Méndez-Couz M. Conejo N. M. González-Pardo H. Arias J. L. (2015a). Functional interactions between dentate gyrus, striatum and anterior thalamic nuclei on spatial memory retrieval. Brain Res. 1605, 59–69. 10.1016/j.brainres.2015.02.005 25680583

[B38] Méndez-Couz M. Conejo N. M. Vallejo G. Arias J. L. (2015b). Brain functional network changes following Prelimbic area inactivation in a spatial memory extinction task. Behav. Brain Res. 287, 247–255. 10.1016/j.bbr.2015.03.033 25813749

[B37] Méndez-Couz M. Conejo N. M. Vallejo G. Arias J. L. (2014). Spatial memory extinction: a c-Fos protein mapping study. Behav. Brain Res. 260, 101–110. 10.1016/j.bbr.2013.11.032 24315832

[B39] Méndez-Couz M. González-Pardo H. Vallejo G. Arias J. L. Conejo N. M. (2016). Spatial memory extinction differentially affects dorsal and ventral hippocampal metabolic activity and associated functional brain networks. Hippocampus 26, 1265–1275. 10.1002/hipo.22602 27102086

[B40] Mishkin M. Ungerleider L. G. Macko K. A. (1983). Object vision and spatial vision—2 cortical pathways. Trends Neurosci. 6, 414–417. 10.1016/0166-2236(83)90190-x

[B41] Mizumori S. J. McNaughton B. L. Barnes C. A. Fox K. B. (1989). Preserved spatial coding in hippocampal CA1 pyramidal cells during reversible suppression of CA3c output: evidence for pattern completion in hippocampus. J. Neurosci. 9, 3915–3928. 10.1523/jneurosci.09-11-03915.1989 2585060 PMC6569931

[B42] Nagayoshi T. Isoda K. Mamiya N. Kida S. (2017). Hippocampal calpain is required for the consolidation and reconsolidation but not extinction of contextual fear memory. Mol. Brain 10:61. 10.1186/s13041-017-0341-8 29258546 PMC5735908

[B43] Nalloor R. Bunting K. M. Vazdarjanova A. (2012). Encoding of emotion-paired spatial stimuli in the rodent hippocampus. Front. Behav. Neurosci. 6:27. 10.3389/fnbeh.2012.00027 22712009 PMC3374936

[B44] Orsini C. A. Kim J. H. Knapska E. Maren S. (2011). Hippocampal and prefrontal projections to the basal amygdala mediate contextual regulation of fear after extinction. J. Neurosci. 31, 17269–17277. 10.1523/jneurosci.4095-11.2011 22114293 PMC3241946

[B45] Packheiser J. Güntürkün O. Pusch R. (2019). Renewal of extinguished behavior in pigeons (*Columba livia*) does not require memory consolidation of acquisition or extinction in a free-operant appetitive conditioning paradigm. Behav. Brain Res. 370:111947. 10.1016/j.bbr.2019.111947 31102600

[B46] Pagani M. R. Merlo E. (2019). Kinase and phosphatase engagement is dissociated between memory formation and extinction. Front. Mol. Neurosci. 12:38. 10.3389/fnmol.2019.00038 30842725 PMC6391346

[B47] Pavlov I. P. (1927). Conditioned Reflexes. London: Oxford University Press.

[B48] Paxinos G. Watson C. (2004). The Rat Brain in Stereotaxic Coordinates-The New Coronal Set. 5th Edn. (Vol. 5) London: Elsevier Academic Press.

[B49] Rescorla R. A. Wagner A. R. (1972). “A theory of Pavlovian conditioning: variations in the effectiveness of reinforcement and nonreinforcement,” in Classical Conditioning II, eds Black A. H. Prokasy W. F. (New York, NY: Appleton-Century-Crofts), 64–99.

[B50] Rueden C. T. Schindelin J. Hiner M. C. DeZonia B. E. Walter A. E. Arena E. T.. (2017). ImageJ2: ImageJ for the next generation of scientific image data. BMC Bioinformatics 18:529. 10.1186/s12859-017-1934-z 29187165 PMC5708080

[B51] Sun Q. Sotayo A. Cazzulino A. S. Snyder A. M. Denny C. A. Siegelbaum S. A. (2017). Proximodistal heterogeneity of hippocampal CA3 pyramidal neuron intrinsic properties, connectivity, and reactivation during memory recall. Neuron 95, 656.e3–672.e3. 10.1016/j.neuron.2017.07.012 28772124 PMC5572758

[B52] Szapiro G. Vianna M. R. McGaugh J. L. Medina J. H. Izquierdo I. (2003). The role of NMDA glutamate receptors, PKA, MAPK, and CAMKII in the hippocampus in extinction of conditioned fear. Hippocampus 13, 53–58. 10.1002/hipo.10043 12625457

[B53] Tamamaki N. (1997). Organization of the entorhinal projection to the rat dentate gyrus revealed by Dil anterograde labeling. Exp. Brain Res. 116, 250–258. 10.1007/pl00005753 9348124

[B54] Taylor J. R. Olausson P. Quinn J. J. Torregrossa M. M. (2009). Targeting extinction and reconsolidation mechanisms to combat the impact of drug cues on addiction. Neuropharmacology 56, 186–195. 10.1016/j.neuropharm.2008.07.027 18708077 PMC2635342

[B55] Todd T. P. Vurbic D. Bouton M. E. (2014). Mechanisms of renewal after the extinction of discriminated operant behavior. J. Exp. Psychol. Anim. Learn. Cogn. 40, 355–368. 10.1037/xan0000021 25545982 PMC4280083

[B56] Vazdarjanova A. McNaughton B. L. Barnes C. A. Worley P. F. Guzowski J. F. (2002). Experience-dependent coincident expression of the effector immediate-early genes arc and Homer 1a in hippocampal and neocortical neuronal networks. J. Neurosci. 22, 10067–10071. 10.1523/JNEUROSCI.22-23-10067.2002 12451105 PMC6758761

[B57] Vianna M. R. Igaz L. M. Coitinho A. S. Medina J. H. Izquierdo I. (2003). Memory extinction requires gene expression in rat hippocampus. Neurobiol. Learn. Mem. 79, 199–203. 10.1016/s1074-7427(03)00003-0 12676518

[B58] Vlachos I. Herry C. Luthi A. Aertsen A. Kumar A. (2011). Context-dependent encoding of fear and extinction memories in a large-scale network model of the basal amygdala. PLoS Comput. Biol. 7:e1001104. 10.1371/journal.pcbi.1001104 21437238 PMC3060104

[B59] Wiescholleck V. Emma Andre M. A. Manahan-Vaughan D. (2014). Early age-dependent impairments of context-dependent extinction learning, object recognition and object-place learning occur in rats. Hippocampus 24, 270–279. 10.1002/hipo.22220 24132946

[B60] Wyss J. M. (1981). An autoradiographic study of the efferent connections of the entorhinal cortex in the rat. J. Comp. Neurol. 199, 495–512. 10.1002/cne.901990405 6168668

